# The geographic evolution of political cleavages in Switzerland: A network approach to assessing levels and dynamics of polarization between local populations

**DOI:** 10.1371/journal.pone.0208227

**Published:** 2018-11-29

**Authors:** Shin Alexandre Koseki

**Affiliations:** Habitat Research Center, École polytechnique fédérale de Lausanne, Lausanne, Switzerland; University of California Los Angeles, UNITED STATES

## Abstract

Scholarly studies and common accounts of national politics enjoy pointing out the resilience of ideological divides among populations. Building on the image of political cleavages and geographic polarization, the regionalization of politics has become a truism across Northern democracies. Left unquestioned, this geography plays a central role in shaping electoral and referendum campaigns. In Europe and North America, observers identify recurring patterns dividing local populations during national votes. While much research describes those patterns in relation to ethnicity, religious affiliation, historic legacy and party affiliation, current approaches in political research lack the capacity to measure their evolution over time or other vote subsets. This article introduces “Dyadic Agreement Modeling” (DyAM), a transdisciplinary method to assess the evolution of geographic cleavages in vote outcomes by implementing a metric of agreement/disagreement through Network Analysis. Unlike existing approaches, DyAM offers a stable measure for political agreement and disagreement—accounting for chance, statistically robust and remaining structurally independent from the number of entries and missing data. The method opens up to a range of statistical, structural and visual tools specific to Network Analysis and its usage across disciplines. In order to illustrate DyAM, I use more than 680,000 municipal outcomes from Swiss federal popular votes and assess the evolution of political cleavages across local populations since 1981. Results suggest that political congruence between Swiss local populations increased in the last forty years, while regional political factions and linguistic alignments have lost their salience to new divides. I discuss how choices about input parameters and data subsets nuance findings, and consider confounding factors that may influence conclusions over the dynamic equilibrium of national politics and the strengthening effect of globalization on democratic institutions.

## 1 Introduction

Debates over new forms of polarization in the electorate stress the importance of assessing how political cleavages evolve over longer periods [1]. For example, recent elections in Europe and North America reflect a new dominant geographic divide between voters: people in core cities and university towns, and those residing in the suburbs and remote communities tend to support opposing political options [2–5]. Bearing resemblance to the so-called “center-periphery” opposition, this divide proves today more resilient than previously dominant alignments among regional and state populations, ethnic communities, urban and rural dwellers, religious groups, etc. [6]. In relation to a growing body of research on the polarization of elites [7, 8] and social media users [9, 10], the complex spatial patterns that emerge from vote outcomes offer insights on many consequential questions: What concerns mobilize the electorate? Where do geographic and political identities intersect? How does context relate to voting behaviors? Can parties and social media influence distant voters? While electoral geography and political science offer insight on those questions, I see two important limitations in existing means to assess the polarization of local populations.

Few methods measure mass partisan polarization, and fewer use vote outcomes [11]. Ideally, the study of political cleavages among voters requires researchers to compare measures taken across time, places, political institutions or any other vote subsets. Yet, current approaches rely on isolated metrics with little statistical reliability. These do not distinguish disagreement from no or low agreement, which seems contradictory to the study of polarization in the electorate. Applications remain largely attached to disciplines when transdisciplinary techniques should allow tremendous innovation. Today, research on the political behavior of individuals, groups and communities remains methodologically disconnected from studies on elites, organizations and states. Likewise, analyses of voters polarization rarely connect with those concerned with online echo chambers or international trade agreements. Yet the fundamental codependence of democratic institutions across levels and the dynamic nature of political processes suggest that the interest for harmonizing methodological and ontological languages between fields remains a major unsolved problem.

Against those limitations, and others I detail below, this article reports “Dyadic Agreement Modeling” (DyAM) as a transdisciplinary method to assess the evolution of political cleavages by implementing a metric of political agreement/disagreement through Network Analysis. The metric I refer to as *Alpha* presents many advantages over common formulas meant to measure correlation between series of vote outcomes. First developed in the context of Content Analysis [12], the *Alpha* accounts for agreement by chance and statistical robustness. As a result, the value space of the *Alpha* offers a quantifiable and theoretical distinction between agreement and disagreement. Moreover, the *Alpha*’s intrinsic control for significance—where extreme values are statistically more reliable than central values—provides a conceptual advantage to the study of collective political behaviors. The implementation of *Alpha* values through Network Analysis gives the research access to several statistical, descriptive and visual tools akin to network approaches, but also to a growing transdisciplinary literature using networks to model human and non-human dynamics. In sum, DyAM holds many advantages in conceptualizing a relational, dynamic, multilevel, multidimensional and multidisciplinary assessment of political cleavages.

In order to illustrate DyAM, I use over 680,000 municipal outcomes of 290 Swiss national popular votes called from 1981 to 2015. With this publicly available data, I produce more than 10,400,000 *Alpha* measures of political agreement/disagreement between municipal voting populations. The frequency and the specificity of Swiss national popular votes allows me to depict the evolution of political cleavages in the country. I divide vote outcomes in four decades, producing corresponding undirected networks in which nodes represent voting populations, and weighted edges represent agreement/disagreement *Alpha* between these populations.

I organize the article as follows: 1) In the next section, I look at the background of DyAM and the transdisciplinary implications of the method for empirical research. I address pivotal and ongoing concerns about mass political polarization and geographic cleavages in electoral geography and political science. Looking at other methodological approaches, I point at the need to measure the evolution of political cleavages between local populations, and the interest of conceiving political disagreement as conceptually distinct from low agreement. Turning to the increasing use of networks to address social dynamics, I make a case for a transdisciplinary application of relational data in order to model mass political behaviors. 2) I begin the “Data and Method” section by giving context to the data that I use to illustrate DyAM—Swiss federal popular votes called between 1981 and 2015. I then describe how to derive *Alpha* measures of political agreement/disagreement from municipal vote outcomes. I conclude the section by discussing two types of networks, which I employ to model the geographic evolution of political cleavages between local populations: undirected weighted graphs and weighted egocentric graphs. 3) I discuss outcomes of the analyses in the “Results” section. The empirical findings corroborate and nuance previous research on the geography of political cleavages in Switzerland, thus providing “real-world” validation to the method. In a first step, descriptive statistics and network metrics show political agreement systematically increased between municipal populations over the last four decades. Cartographic and graph representations of the networks’ communities illustrate the clear evolution of political divides between local populations. In the 1980s, voters were clearly fragmented in multiple regional and micro-regional clusters. In the 1990s, boundaries between linguistic communities dominate the network, thus confirming the idea of an ethnic cleavage—the so-called “Röstigraben”—in Swiss politics. This divide dissipates gradually, however, in the 2000s when voters in German-speaking cities and those across French-speaking municipalities agreed increasingly over vote outcomes. This transition culminates in the 2010s when municipal populations split into three groups: “introverts” German-speaking villages in the remote part of the country; “selective” German-speaking suburbs; and “extroverts” an alignment of German-speaking cities, towns and villages with a historical presence of tourists, and of French-, Italian- and Romansh-speaking minorities. I discuss the implication of such classification for the Swiss political landscape, and in relation to seemingly similar dynamics in other Northern democracies. Egocentric Network Analyses give further information on shifts in political disagreement between parts of the country. I use three examples to show how municipal populations that most disagree with each other behave over time: “extrovert” Geneva and Zurich, on the one hand, and “introvert” Unteriberg, on the other hand. Here, I project *Alpha* values directly on choropleth maps of Swiss municipalities to illustrate the relative position of those “extreme case studies” in the last four decades. 4) In the light of these results, I discuss the contribution of DyAM as a transdisciplinary method to investigate the geographic evolution of political cleavages between local populations. I explore how choices about the case study, input parameters and data subsets nuance findings made with DyAM. To contextualize outcomes of the analysis, I consider confounding factors that may influence conclusions over the strengthening effect of globalization on political attitudes in Switzerland. I list the methodological advantages of DyAM over other approaches, and highlight how the method grounds an empirical definition of political polarization of the electorate. While Swiss direct democracy may provide an ideal case study, the method’s true potential surely locate in its ability to offer multi-dimensional, multilevel, dynamic and comparative analyses. I conclude this article by briefly pointing at the many ways further methodological developments and empirical applications of DyAM can contribute to enriching a transdisciplinary research on political behaviors.

## 2 Background

### 2.1 From political divides to polarization

Shifts in the traditional divisions of partisan support constitute a hallmark of contemporary democracies. Traced back to the beginning of the Twentieth-Century, early signs of new cleavages among voting populations first appeared in Europe and rapidly extended across Northern nations [13–15]. For well over a hundred years, industrialization, secularization, nationalization, urbanization, globalization and digitalization shape the attitude of voters and their relationship with political elites.

Many studies highlight the geographic dimension of new political cleavages in the population [16–18]. In their pivotal work on conflicts and their translation from and into party systems, Lipset and Rokkan [19] show that divergent attitudes between culturally dominant and subjected populations, workers in primary and secondary economic activities, and supporters of established and subversive values generate more or less salient divides between voters. The overlap of cleavages and the spatial cluster of voters produces local and regional contrasts in partisan supports, macroscopic patterns in vote outcomes and a geographic institutionalization of conflicts [20].

The work by Lipset and Rokkan opened new considerations on the ecology of political behaviors across geographic levels [21]. Among others, researchers identify “nationalization” [22] and “metropololitanization” [23] as new mechanisms that translate voters’ conflicts into party systems. Developing experimental approaches to shed light on political behaviors, Enos establishes empirical evidence on the effect of segregation [24], interaction [25] and ground campaigning [26] on vote outcomes.

Political conflicts define normal democratic processes [27]. Lasting and deepening cleavages, however, could indicate an alarming phenomenon: political polarization. Although widely discussed in the media and in the scientific literature since the early 1990s, the argument over an increasing regional divide in political attitudes remains largely unsettled [28]. Sartori [29] originally describes political polarization in terms of “ideological distance” between voters or groups of voters. Di Maggio adds, however, two conditions: polarization occurs when “opinions on an issue are opposed in relation to some theoretical maximum [and] the increase in such opposition over time” [30][p. 693]. Following this logic, Fiorina identifies conceptual and methodological weaknesses of several studies on polarization in the US public and shows the appearance of mass cleavages results from strong divides in the country’s two-party system [31–33]. He debunks prominent analyses based on attitudinal surveys [34] and argues that vote reports, election returns, or approval ratings “cannot be used as evidence of polarization—for or against” [31][p. 556].

The study of political polarization requires us to measure the intensity and the evolution of cleavages among voters, and the possibility to cross-validate results across geographic levels, democratic institutions and types of data [1]. The method I present in this article makes a step in that direction by offering two significant contributions: the possibility to measure and compare agreement and disagreement between voters or groups of voters across time, and a methodological framework for a transdisciplinary research on mass polarization.

Previous literature on geographic political cleavages assumes that local shares of partisan votes capture polarization dynamics [5, 35–37]. Yet, punctual and strengthening support in a given location does not necessarily entail that polarization occurs at the national level. On the other hand, measures of national polarization offer no insight on the spatial or social clustering of political attitudes [11, 38].

Similarly, many scholars point at the necessity to better investigate the link between polarization in the political elite and polarization of voters [11, 33, 38, 39]. Following Rice’s measure of elite cohesion using roll call votes [40], numerous formulas were proposed to measure representativeness, lines of conflict and the cohesion of groups in parliaments [41–44]. These metrics display, however, important weaknesses that are shared by many commonly used coefficients of correlation [45]. They apply exclusively to two-option votes and can’t accept missing data. They only produce scores for one vote at the time, rely on arithmetic mean values for more than one vote, and therefore can’t control for randomization or agreement by chance. Their value space ranges from 0 to 1, which creates confusion between low agreement and active disagreement—a technical limitation with meaningful conceptual implications, especially for the study of politics. Their statistical properties remain vaguely known and have been proved to be unreliable [46–48].

Few studies use quantitative metrics to assess political cleavages and cohesion in the population. Those that do rely on attitudinal surveys or outcomes of popular votes [49, 50]. The advantage of using data from the latter is manifold. Like electoral reports, outcomes of popular votes directly reflect people’s behavior at the poll, and conclude *sensu stricto* the political decision-making process. Whether direct or consultative, popular votes entail simpler issues and better frame what is at stake. Like survey questions, popular votes offer precise insights on a population’s attitude towards specific matters. While most geographic work on popular votes employ a case-by-case approach and visual heuristics to determine local and regional contrasts in the political attitudes, computational tools have also opened up the field to a new kind of analysis.

### 2.2 Political cleavages in Switzerland

The Swiss direct democratic system provides reliable material to analyze political cleavages within the national population. Very frequent votes on a wide array of topics and publicly accessible data make Switzerland a perfect “laboratory” for the study of mass political behaviors [51]. Looking at vote outcomes of local and regional populations, researchers have examined where dominant political cleavages appear in the country using multivariate analysis techniques [52–57].

For example, Diskin and colleagues [54] analyze cantonal outcomes of 537 federal popular votes called between 1866 and 2005 and find that collective political behaviors across cantons become more and more similar with time. In discussing their results, the researchers suggest that popular votes at the national level have themselves contributed to the ideological alignment of regional populations. In a similar fashion, Hermann and Leuthold [56] recode local outcomes of federal popular votes to reveal three dimensions that structure the Swiss political landscape: liberal/conservative, left/right and ecological/technocratic. They then combine their factor analysis to experimental cartographic methods and highlight the role of geographic characteristics on the political behavior of local communities. In this research, they show the dividing effect of language, the clustering effect of urbanization and the disruptive effect of local social contexts onto the political dimensions of Swiss popular votes [52].

In order to identify such structuring dimensions in the data, both teams of researchers rely on multivariate analyses to maximize the variance of correlated variables and generate uncorrelated vectors, or N-dimensions that describe the data [58]. While this approach may provide insightful observations, it has several important limitations for the study of political cleavages and polarization. Every transformation is self-referential, which means that the projection of populations onto dimensions is relative to the value space of input data. Moreover, the operation does not accept missing values in input variables, and dropping a single entry greatly affects output vectors. It is therefore impossible to meaningfully measure the ideological distance between populations or compare data subsets such as sectional periods, political themes or types of vote. In order to overcome these limitations, Dyadic Agreement Modeling (DyAM), which I present in this article, adds to the recent redevelopment of Network Analysis in political research.

### 2.3 Exploring political cleavages with Network Analysis

The relational nature of power makes Network Analysis an ideal approach to study political dynamics [59]. Over the last fifteen years, new computational methods in network research provide means to answer pressing and important questions in the study of political behaviors, democratic institutions, policies and international relations. Against the “methodological individualism” that prevails in political and sociological research, networks hold a unique capacity to model complex systems. More than a mere method, networks analysis results from a new paradigm that posits and explores political phenomena as resulting from relationships between agents [60]. In network research, those agents are called nodes, and the relationships that tie them together are called edges. Hence, by looking at the organization of those nodes and edges, many attributes of networks translate directly or indirectly into parameters and characteristics of “real-world” political organizations.

Among the most common of those parameters, *density* describes the degree of connectivity between nodes of the network. Mathematically, it is defined as the ratio of edges in a network divided by all possible edges that could exist between its nodes. In order to express the unequal distribution of density across the network, *modularity* measures how it is fragmented into communities. High modularity means a network has dense connections between certain nodes but sparse connections between groups of connected nodes. In certain networks, another important parameter is their degree of *transitivity*. Also called “clustering coefficient,” the global measure of transitivity corresponds to the number of closed triplets divided by the number of closed or open triplets in the network, where triplets are three nodes connected by either three (closed) or two (open) edges. Transitivity is an important characteristic of real systems, either social or natural. Together with modularity, transitivity shows agents connect with other agents through shared relationships, rather than randomly.

The community structure of the network represents the primary characteristic of real systems [61]. In such networks, communities (or modules) consist in groups of nodes that have more edges between each other and comparatively fewer edges joining nodes in other groups. The presence of communities within a network can be detected by measuring its modularity. Evidently, defining limits or overlaps between communities is of great importance for empirical research [62]. Despite the huge effort of a large interdisciplinary body of scientists working on the detection of community structures, the problem has no satisfactory solution. Instead, different techniques are used in relation to parameters and attributes of the networks, and offer different advantages and disadvantages to the detection of communities [63].

A common application of community detection techniques in the study of political dynamics looks at the groups and divides that animate legislative processes [64–66]. In this literature, researchers look at the sponsorship and co-sponsorship of bills by individual parliament members using roll-call data. Such application of Network Analysis to political research offers means to understand power relationship as non-hierarchical systems, and how those systems can adapt to changes in partisan support [7].

Although marginal, the application of Network Analysis to voters’ behaviors [67, 68] demonstrates how democratic decision-making is tied to contexts and interdependence between voters. Those works explore the “micro-macro” divide in politics, thus reiterating a long-standing claim of political geographers on the importance of considering the political relationship between places, groups and individuals, what Agnew [6] calls the “spatial situatedness of human action in contrast to the non-spatial sorting of people out into categories based on census and other classification schemes that inspires most conventional social science.” This article introduces Dyadic Agreement Modeling as a technique to assess the evolution of political cleavages between local populations, thus offering a contribution to the study politics as a relational and situated social process.

## 3 Data and methods

This study uses municipal outcomes of Swiss federal popular votes, which are central to the legislative and constitutional systems in Switzerland. Because of their frequency, votes called in the Swiss direct democracy provide an ideal case study to examine the evolution of political cleavages based on agreement/disagreement between local populations. Results I report in section 4 of this article stem from a network analysis of almost 2,300 municipal populations and more than 2,600,000 measures of political agreement/disagreement between them.

Switzerland is a federation of twenty-six highly autonomous states called “cantons.” Each canton has it own constitution with a parliament that enacts laws within its jurisdictions. Cantons themselves federate even smaller state-like municipalities called “communes.” Communes, which cover the entire national territory, have their own governmental assemblies to oversee local affairs. Swiss nationals’ citizenship is bound to the country, but also to a commune, and to the canton in which the commune is located. This three-level citizenship is conditional to the nationals’ right to take part in popular votes that are called for several times a year following a principle of direct democracy implemented at all three governmental levels [69].

Since the adoption of its modern federal constitution in 1848, Switzerland has called over six hundred national popular votes, half of which have taken place since the 1980s. In this direct democratic system, citizens hold the ultimate decision-making power over any change to the law and the Constitution of the country. Federal popular votes concern all aspects of legislation attributed to the federal parliament: political functioning, foreign policy, security policy, economy, public finance, infrastructure, planning and the environment, social policy, as well as education, culture and communication. On a polling day, voters accept or reject between one and six different federal vote objects, each concerning a different change to the law or the Constitution. Votes are largely discussed in the media, and the government, major parties and *ad hoc* support and opposition groups advertise their positions to the public during the months that precede voting day. From a legal point of view there are three types of votes objects: Obligatory referenda by which the population is required to accept or reject a modification to the federal Constitution proposed by the Parliament; facultative referenda, which take place when the population petitions the Government against the enactment or the amendment of a law; and popular initiatives that can be called for when a certain share of the population requests a modification to the Constitution. Under certain circumstances, the federal government may propose a counter-project to a popular initiative [70].

Actual poll data on federal popular votes are available at different levels of aggregation through the online portal STAT-TAB of the Swiss Federal Office of Statistics (SFOS). Data at the municipal level provides the finest resolution but is available only for votes called from June 14, 1981, onward. The data reflects official ballot counts following each vote day, which take place nationwide four times a year, at predetermined dates. Voters—Swiss nationals over 18 years of age—cast their vote at a local polling office within the municipality where they have registered their main residence. They may also send their vote by mail to the local polling office where they are registered one day at least before voting day. By contrast, the VOTO dataset (formerly VOX) is derived from a nationwide survey of roughly a thousand Swiss voters, and is conducted after each voting day since 1977. The dataset comprises entries on respondents’ votes, individual characteristics, family setting and other contextual information. VOTO surveys provide no information on the geographic allocation of respondents, beyond the national language in which they prefer to answer (German, French, Italian or Romansh), and respondents’ own report on the type of urban environment in which they live (remote area, countryside village, small town, larger city).

VOTO data fit better the analysis of polarization among the population in relation to individual psychological characteristics and sociological attributes, rather than a geographic analysis of collective polarization, like I report here. Such dataset, like municipal vote outcomes during elections and related SELECT surveys, could be deployed to conduct complementary analyses that address a wider spectrum of political behaviors. In this article, I limit myself to an assessment of the geographical clustering of political agreement between voting municipal residents.

### 3.1 Federal popular votes 1981-2015 datasets

The dataset I use concerns Swiss municipal poll stations for 290 federal popular vote objects called between June 14, 1981, and June 14, 2015. In order to assess the evolution of agreement between voting municipal populations, I divide the dataset in four sectional periods (1981-1990, 1991-2000, 2001-2010 and 2011-2015) that correspond to three calendar decades and the first half of the current calendar decade. For each period, I report results that comprise all juridical types of votes and political themes.

The SFOS proactively aggregates the data to 2,291 statistical area units that correspond to the 2,324 municipal boundaries at their status on January 1, 2015. The SFOS combines some smaller municipalities in single statistical areas to ensure anonymity, and re-aggregates the data following the institutional transformation of area units, such as municipal mergers. In the data, municipal vote results are expressed in a three-decimal percentage and represent the proportion of valid ballots in favor of the vote over the number of valid ballots cast in the municipality. I find nine municipalities without data for the entire period, and six municipalities without data for votes called before 1997; those missing values are probably due to an error in the original compilation of the data, so I remove those fifteen municipalities from the analysis altogether, leaving 2,275 municipal populations. In order to compute the metric of agreement/disagreement between those groups of voters, I recode the 659,750 municipal vote results to binary vote outcomes following the majority rule of fifty percent plus one. I explain the choice of binary outcomes over percent support in the next section.

### 3.2 Computing political agreement/disagreement

To overcome the limitations of computing agreement between groups of voters, I introduce the use of a measure of agreement/disagreement exogenous to research on political cleavages: Krippendorff’s *Alpha*, a formula that was developed in the context of content analysis, and that is ideal for quantifying agreement and disagreement in vote outcomes for several reasons outlined below.

The measure corresponds to the degree to which a number of entities provide agreeing/disagreeing answers over a series of specific questions. In its original context of reliability assessment, Krippendorff [71][p. 113] refers to this as “sameness or difference between two values that are separately attributed to the same object.” Variations of the *Alpha* accept many value types: nominal, ordinal, interval, ratio, polar and circular. I take this approach with Swiss direct democracy by using municipal outcomes over federal popular votes. I use binary vote outcomes to facilitate the algorithmic computing of the measure of agreement/disagreement. The *Alpha* thus produces a reliable relational measure of political agreement and disagreement between all possible pairings of municipal voting populations, generating edges for the network approach I implement.

In order to operationalize this metric of agreement/disagreement, I employ a case of the *Alpha* for nominal values and two observers with missing data. In Eq (1) *D*_*o*_ is a measure of the observed disagreement and *D*_*e*_ is a measure of the disagreement that can be expected when chance prevails; *A*_*o*_ is the observed agreement, *A*_*e*_ is the expected agreement, *A*_*max*_ is the largest possible agreement, *n* is the total number of objects voted upon, *o*_*cc*_ is the number of observed coincidences, and *e*_*cc*_ is the expected number of observed coincidences with *m* number of municipal populations concerned [12, 72]. Given the large number of possible pairs of municipal voting populations, I wrote a Python script that computes the *Alpha* of each pair for selected vote subsets.

$$\begin{array}{c} \alpha_{\alpha \in [-1,1]}^{m>2}=1-\frac{D_{o}}{D_{e}}=\frac{A_{o}-A_{e}}{A_{max}-A_{e}}=\frac{\sum_{c}o_{cc}-\sum_{e}e_{cc}}{n-\sum_{c}e_{cc}}=\frac{\sum_{c}(o_{cc}-e_{cc})}{\sum_{c}(n_{c}-e_{cc})} \end{array}$$ (1)

The *Alpha* has several qualities that make it an ideal tool for measuring agreement and disagreement among voting populations in the context of popular votes. Unlike many other measures of political congruence that assess sameness of attributed values between no agreement and perfect agreement (thus ranging from zero to one), the *Alpha* assumes a definite value for agreement/disagreement by chance at zero. The intrinsic control for agreement/disagreement by chance means that values above zero quantify the degree of agreement between two given populations, while values below zero quantify the degree of disagreement. Perfect agreement is measured at one, while perfect disagreement is measured at minus one. The equation can generate “not a number” value in the hypothetical situation where two populations produce the same single type of outcome (all “yes” or all “no”). Another advantage of the *Alpha* is its resilience to the number of votes considered. In the context of the analysis I report, this enables me to compare *Alpha* values across periods that contain a different number of votes [71][p. 113].

Chiefly, my objective is to measure the degree of political congruence between municipal voting populations. The use of the *Alpha* differs in this context from content analysis where it measures reliability between coders motivated by perfect agreement. For this reason, I do not define a threshold of necessary agreement but more directly use the *Alpha*’s intrinsic control for agreement by chance and value range as a proxy for political congruence. This allows me to develop a relational approach of geographic analysis free from topographical constrains and suited to a comparison between vote subsets.

### 3.3 Towards network approach to the geography of political cleavages

Network Analysis provides a suitable paradigm and methodological framework to look at the similarity between distant places. The approach focuses on relational ties while maintaining the integrity of the units of analysis. In this present implementation of Network Analysis, I build a series of graphs to assess the political congruence between local populations. I generate each network solely with the degree of agreement that local populations reach over a series of popular votes. I therefore built these networks independently from the physical distance between the populations. From the graphs, I first identify “political communities” as groups of municipal populations that agree more with each other than with other units of analysis. To do so, I only consider (positive) agreement values between populations. In a second step, I illustrate how (negative) disagreement values can be considered by assessing agreement/disagreement values from the egocentric networks of selected local populations. The following section outlines how I implement measures of political agreement between voting municipal populations with Network Analysis and how I identify those political communities using a network community detection algorithm.

Let *W* denote the agreement/disagreement matrix, where the component *W*_*ij*_ ∈ [−1, 1] is the degree of agreement/disagreement that the voting municipal population *i* has with one other municipal voting population *j*. This corresponds to an undirected weighted complete graph with municipal voting populations represented as nodes and agreement/disagreement values as edges between nodes. Agreement *W*_*ij*_ over binary outcomes between the voting majorities of two municipal populations is measured with the *Alpha*. From *W*, I derive *W*′, an agreement matrix, where the component *W*′ ∈ [0, 1] masks the value space above the definite value of agreement by chance. This second matrix corresponds to an undirected weighted graph in which edges correspond to (positive) agreement values between voting populations.

By treating municipal vote outcomes as a network consisting of nodes (municipal voting populations) and edges (political agreement between populations), I can test whether the organization of network communities is coherent with empirical observations on the geography of political cleavages between local populations in Switzerland. To this end, I manipulate the data with the “igraph” package for Python [73]. The package offers several algorithmic solutions to detect community structures in weighted graphs. Faced with the difficulty of selecting the most appropriate algorithm, I choose to use the Newman leading eigenvector “top-down” method and let the algorithm define the number of communities based on the modularity of the original network and edge weight. The method is especially suited for detecting communities in very large graphs (*N* > 500) with a high density of edges between nodes (*D* >.5) [74–76].

Dyadic Agreement Modeling provides an unprecedented attempt to jointly apply the *Alpha* measure of agreement/disagreement and Network Analysis to political vote outcomes. Further studies concerned with the mapping of political communities could therefore benefit from the rich declensions of both methods, which together offer robust conceptual and statistical properties, and potential applications in many different contexts. For the present study, it is important to note that the algorithm detects resulting communities independently from the geographic allocation of voting municipal populations or any other geographic or demographic characteristics of those populations. The patterns that emerge from the community detection reveal solely the political congruence of concerned populations. In the specific test case of Switzerland, the resulting geography can therefore freely express regional and inter-regional political cleavages and alignments and illustrate ideological divides among Swiss people today. Communities in the network should therefore align with empirically established geographical divisions such as historical regions, linguistic communities, religious groups, cantons, and urban-rural or center-periphery oppositions.

## 4 Results

In this section, I present how DyAM reveals the dynamics of political cleavages between Swiss local populations. The effective combination of Network Analysis to a measure of agreement/disagreement over municipal vote outcomes offers persuasive alternative technique to multivariate approaches using similar data [52–55]. The first part of the results confirms the potential of network-based method to depict the political landscape of local populations as well as its transformation. Overall, metrics of agreement/disagreement suggest local populations are increasingly congruent over political issues. When looking into how this landscape is organized, an analysis of all votes called over recent decades supports the idea of strong regional alignments and clear divides between linguistic communities [77, 78]. This finding is, however, put into perspective when examined against more recent outcomes; agreement networks that include votes called in the 2000s and 2010s point to a new layout of political cleavages between local populations. Metrics from those networks show a constant increase in political agreement between local populations and linguistic groups. This supports findings I make from graph visualizations on the nationwide convergence of municipal vote outcomes.

In the second part of the results, I show another use of DyAM, which consists of the cartographic transposition of the networks. Keeping with a combination of visual heuristic and network metrics, I map out the political communities detected in networks on population maps of Switzerland. I then fine-tune the colorimetric rendering of the population clusters to afford a sharper reading of the geographic evolution of political cleavages. This second step facilitates a linkage of the relationship that ties changes in the political divides of local populations to their geographic and demographic characteristics. Here the results speak for themselves. Once transposed to maps, network communities underpin a recent alignment between physically distant populations, and support the idea of a topological organization of the Swiss political space. In keeping with recent observations, cartograms illustrate how global connectivity has become a dominant source of political differentiation between local populations.

In the last part of the results, I reintroduce (negative) disagreement values in DyAM by illustrating the use of egocentric networks with the municipal populations of Geneva, Zurich and Unteriberg. For each of these examples, I consider agreement and disagreement values obtained with all other local populations over four sectional periods. I then visualize those values with choropleth maps of Swiss municipal boundaries. This additional implementation of the method allows me to address the political transformation of 2,275 local populations as specific case studies. While each of the three examples I report tells a different story, agreement and disagreement values confirm overall tendencies I describe in the previous parts of the section. Among other findings, I note the increasing agreement between voters residing in large cities, their political alignment with linguistic minorities, and the resilient divide between those populations and German-speaking communities in suburban and remote areas.

Overall, the results support the accuracy and utility of DyAM to study the evolution of geographic cleavages between local populations. Many of the macroscopic patterns I find concur with previous research on Swiss political cleavages. Others match observations that have been hinted at but not empirically demonstrated. In comparison with other approaches, DyAM offers a stable, resilient and precise method to assess political congruence between local populations. Moreover, its fixed measure of agreement/disagreement provides a timely and necessary technique to address polarization as a dynamic phenomenon. The *Alpha*’s intrinsic control for agreement/disagreement by chance, its acceptance of missing values and its statistical robustness make it an ideal metric of political congruence. On a conceptual level, the *Alpha* stands closer to the idea of political agreement/disagreement than other metrics. Its implementation into networks allows for a direct and coherent interpretation of multiple network parameters. Moreover, by fitting in the scheme of network approaches, DyAM opens up to multiple transdisciplinary tools to assess political dynamics statistically and heuristically.

### 4.1 Mapping political agreement between local populations

**Fig 1:**
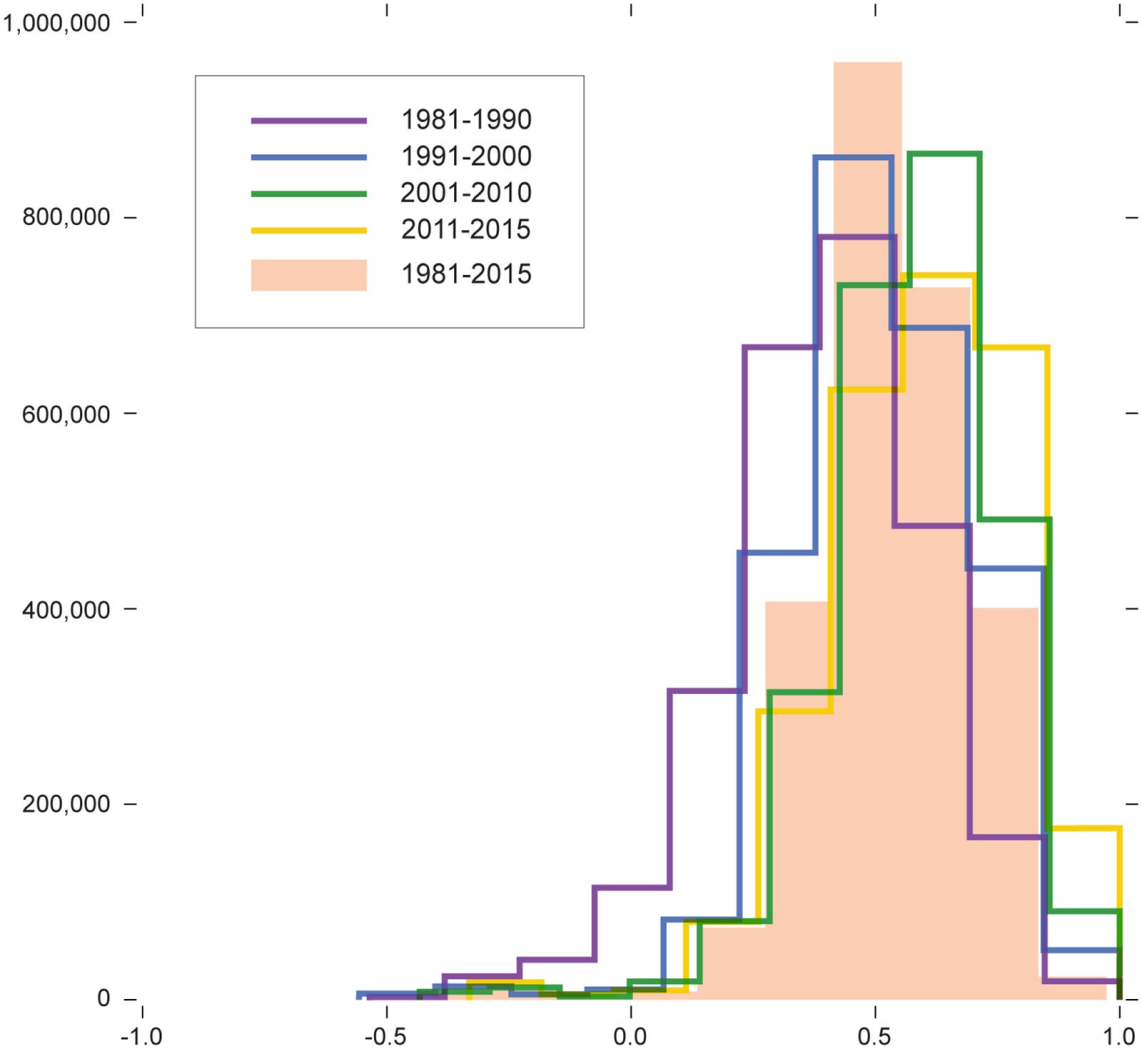
Measures of agreement/disagreement between Swiss municipal populations of voters.

In the *Alpha*’s original context of Content Analysis, it makes sense to define a minimum agreement value under which coders are considered unreliable. This is because coders should aim for perfect agreement [79][p. 86]. For the study I report here, perfect agreement between municipal populations is not desirable. Perfect consensus between groups of voters hardly illustrates a functioning democratic system, nor does political agreement by chance, which translates into *Alpha* values close to zero. I start by asking what level of political agreement/disagreement between municipal populations can be expected from a political institution such as Swiss direct democracy. Fig 1 and Table 1 help answer this question. The former shows distributions of *Alpha* measures of agreement/disagreement between every pair of municipal populations for each period I report; the latter is concerned with characteristics of those distributions.

**Table 1:** Characteristics of agreement/disagreement between Swiss municipal populations of voters.

	1981-1990	1991-2000	2001-2010	2011-2015
Maximum	1.000	1.000	1.000	1.000
Median	0.419	0.510	0.598	0.609
Mean	0.409	0.516	0.586	0.602
Minimum	-0.453	-0.461	-0.400	-0.319
Standard dev.	0.196	0.175	0.162	0.177
Missing *Alpha* values	43339	20574	20574	22855
% Negative values	2.398	0.339	0.138	0.070
% Populations disagreeing	97.905	96.857	49.280	28.416

As one could have expected, political agreement between local populations has little to do with chance, and large shares of municipal populations agree with each other. The figure also shows to which extent certain populations disagree on most political matters. Although I find political agreement to be more frequent, political disagreement between voting populations also exists. Depending on the period concerned, between 28.41% and 97.90% of voting populations disagree with at least one other population. The share of disagreement values represents between 0.02% and 2.40% of all *Alpha* measures. In both cases, those proportions decrease over time as the average agreement between local populations improves from one sectional period to the next. Wilcoxon signed-rank tests on paired distributions confirm the significance of each increase.

The plotting of agreement/disagreement value distributions for four consecutive sectional periods already highlights the political dynamic between Swiss local populations, and gives us a general sense of the evolution of the nation’s political landscape. Another way to look at this data is to articulate agreement values between voting municipal populations into networks of municipal populations, thus opening the analysis to a new set of metrics and visualization techniques. In Table 2, I report characteristics of those weighted networks for the entire data and for each four sectional periods. Those network metrics underscore additional information on the geographic evolution of political cleavages in the Swiss population.

**Table 2:** Characteristics of the Swiss agreement network.

	1981-1990	1991-2000	2001-2010	2011-2015
Density	0.959	0.989	0.991	0.991
Modularity	0.075	0.075	0.072	0.078
Transitivity	0.981	0.999	0.999	0.999

Federal popular votes address a whole range of political topics regulating several aspects of Swiss society. Perhaps this explains why voting populations tend to be consensual. When considering positive *Alpha* values, Swiss municipal populations form single connected networks. Increased density from one sectional period to the next suggests some structural changes in the networks as more paired populations that disagreed in previous decades later agree with one another (although the statistical uncertainty for *Alpha* values closer to zero nuances this observation). Moreover, analyses of more precise subsets of the original data show the apparent increase in political agreement is mostly due to *popular initiatives* and *facultative referenda*. For *obligatory referenda*, agreement between paired populations increases over the first thirty years, but drops for votes called between 2011 and 2015. While the resulting network for this period is much sparser (*D* = 0.31, against *D* = 0.99 for 2001-2010), it also has the highest average *Alpha* value (*α* = 0.76, against *α* = 0.67 for 2001-2010). Those values suggest a strong polarization of local populations could have occurred, opposing populations that support the Parliament’s legislative work, and those that strongly oppose it. Considering all votes again, slight changes in modularity and global transitivity confirm this structural change in the Swiss political landscape. The two metrics indicate agreement increases inside groups of populations that strongly agree together but might decrease between these groups. In the present case, such metrics must be considered carefully because high density and transitivity might reflect properties of political agreement; if one population highly agrees with two other populations, chances are those two also highly agree with each other. In order to get a better understanding of those structural changes in the networks, I turn to visual approaches.

**Fig 2:**
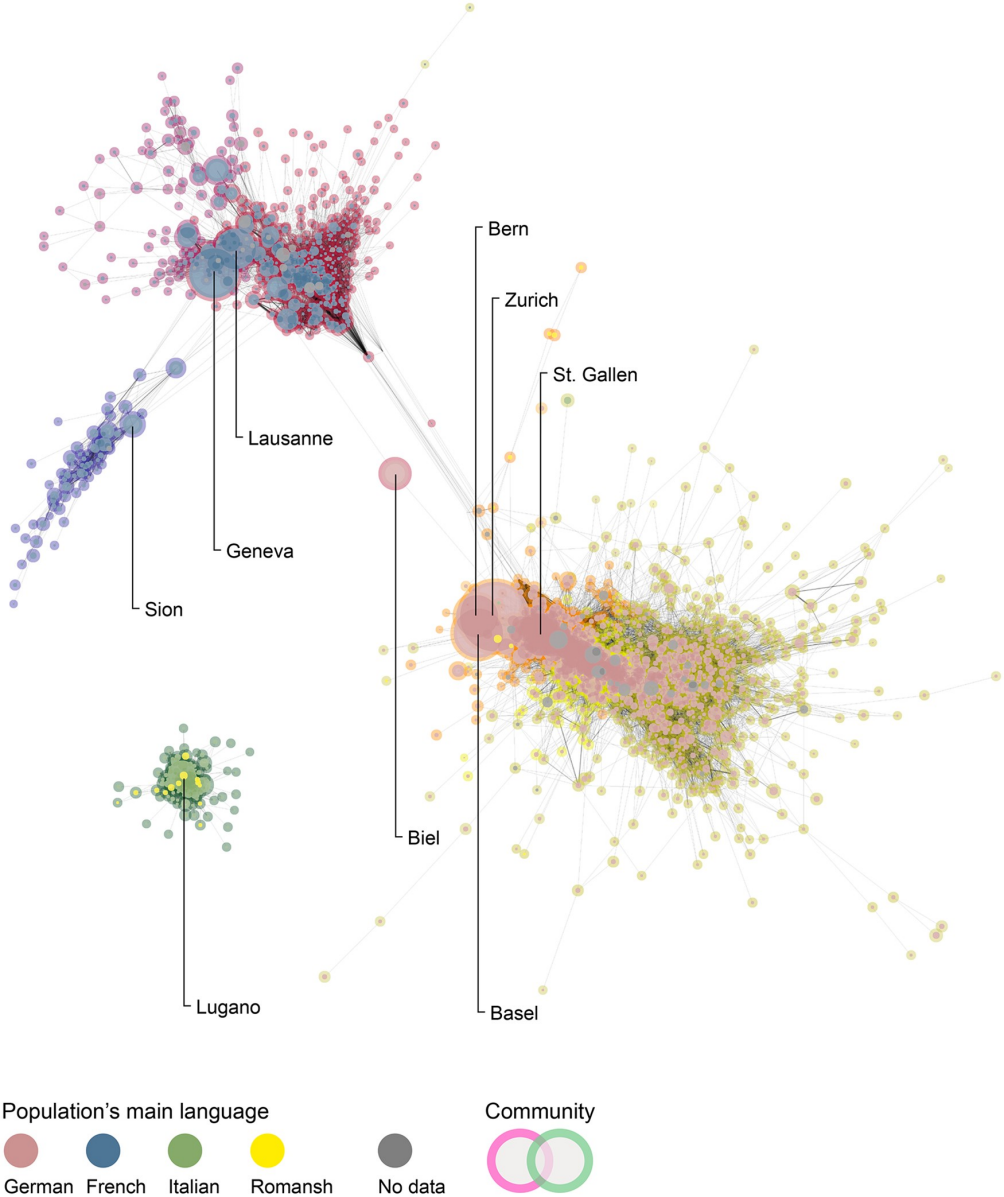
Political agreement above 0.8 *Alpha* for votes called between 1981 and 2015. Edges’ width is proportional to *Alpha* value. Nodes’ size is proportional to residing municipal population, status on 1 January 2000 for municipal boundaries on 1 January 2015. Population’s main language is most spoken national language, status on 1 January 2000 for municipal boundaries on 1 January 2010. No language data for municipalities merged after 2010.

The heuristic use of graphic outputs has had a historic place in the analysis of networks, accounting as essential to theoretical modeling and empirical testing [80]. Findings made this way are, however, very dependent on characteristics of networks and parameters used to generate their visualization. To remain readable, visual graphs differ from their mathematical counterparts by limiting the use of certain attributes. For instance, it is difficult to represent edges with a negative weight—although they are common in real world social networks—because such values are incompatible with most spatialization algorithms [81]. Yet graphic outputs of nodes and edges generate more tangible and convincing results. They also enable researchers to engage with intuitive reasoning and associations hardly reducible to variables. In Fig 2, I engage in such an approach by removing agreement values above the threshold of *α* = 0.8, which I find generates the most readable visualizations for the networks I report. As there is a larger uncertainty for values closer to *α* = 0, the resulting graphs should better translate the cognitive reading of political cleavages. Moreover, using a value well above the average measure of agreement supports the view that in politics, looking at a strong agreement is more relevant than looking at weaker one.

When considering strong agreement over all votes cast since 14 June 1981, I find that municipal populations with the same language cluster in tight-knitted groups. Most French-speaking and German-speaking municipal populations do not agree much on political matters. Instead, these linguistic groups form quasi-independent components and remain almost fully isolated from each other. Yet bilingual municipalities, larger cities and some affluent towns tie the two linguistic groups together, keeping most of the country’s local populations within a single continuous system. The use of community detection on the network reveals that smaller and tighter political communities compose larger and seemingly united groups. Hence, German-speaking populations form six political communities that comprise between *n* = 2 and *n* = 648 municipal populations. Likewise, French-speaking populations cluster into three main communities made up of *n* = 62, *n* = 108 and *n* = 375 municipal populations with varying demographics. As for Romansh and Italian-speaking municipalities, the former side with German-speaking populations only, while the latter separate from the main component of the network and form a fully independent entity. The political divide between Swiss populations mostly follows the linguistic frontiers that characterize the country’s landmark cultural diversity. These results appear to buttress the popular concept of a cultural clash between German-speaking and French-speaking populations, known in Switzerland as the “Röstigraben” [82, 83]. In the following section, I report the sectional analysis and show that political cleavages in Switzerland are not static. What appears to be a trenchant ideological split has begun to reverse.

**Fig 3:**
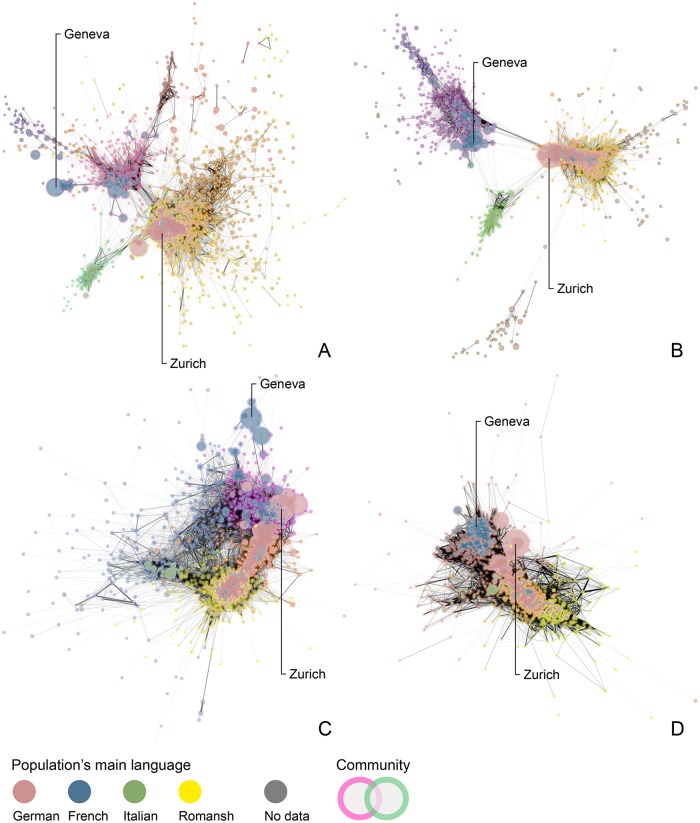
Political agreement above 0.8 *Alpha* for four sectional periods between 1981 and 2015. (A) 1981-1990. (B) 1991-2000. (C) 2001-2010. (D) 2011-2015. Edges’ width is proportional to *Alpha* values. Nodes’ size is proportional to residing municipal population, status on 1 January 2000. Population’s main language is most spoken national language, status on 1 January 2000. No language data for municipalities merged after 2010.

Political cleavages between local populations have undergone important transformations between 1981 and 2015; in Fig 3, DyAM illustrates the changing political landscape of Switzerland with the main network components of the four sectional periods. In the 1990s, a clearer divide appears between German-, French and Italian-speaking populations. Within each of these groups, the density of edges between nodes increases as two subgroups form. A decade later, political agreement between local populations continues to increase within and between linguistic groups. During that period, some French-speaking populations align with a group of larger German-speaking cities, tying the two linguistic regions together within common political communities. On the opposite side, other German-speaking populations develop stronger agreement with Italian speakers, which in turn, connect to smaller French-speaking municipalities. This phenomenon intensifies in the following sectional period, which also sees the nationwide political convergence of local populations. Over the last five years, populations that belong to the French-speaking and Italian-speaking groups form a single political community with larger German-speaking cities. The two remaining political communities are composed entirely of German-speaking populations, which highlights the resilience of cultural characteristics in shaping the current political landscape of Swiss direct democracy.

The relational approach, which I present here, provides insightful knowledge on a society’s political transformations, as it allows researchers to examine social spaces free from the constrains of traditional topographical proximity. Overall, findings from the agreement networks show a partitioning of political communities can be achieved using only political congruence between local population. In the next section of the article, I explore the cartographic mapping of networks’ community structures to shed light on what kinds of regionalization patterns and geographic cleavages can be expected from political agreement. Resulting maps suggests a strong influence of global connectivity on the current state of political dynamics in Switzerland. Those findings support similar observations across other national contexts, and illustrate how the evolution of political cleavages in the population follows global trends [84].

### 4.2 Towards new geographic divides

Research using network approaches can highlight the role of geographic contiguity on spatial practices and behaviors [85]. Such work supports a generalized view in geographic literature according to which near things are more related than distant things [86]. Nuancing the equation of distance to Euclidean metrics, Lévy reminds us that humans have developed different ways of engaging with space, and that it should therefore be measured accordingly [87]. Because most studies focus on the making and the maintenance of interaction through space, I seek a different type of spatial relationship. Political congruence between local populations is a critical social dyad to address in today’s context. The practice of referring to populations’ political alignments and cleavages plays a major role in democratic processes, as well as in social dynamics. In this section, I look more closely at the geography of those alignments and cleavages and their evolution through time by exploring the spatial dimension of network characteristics I produce in the previous section.

**Fig 4:**
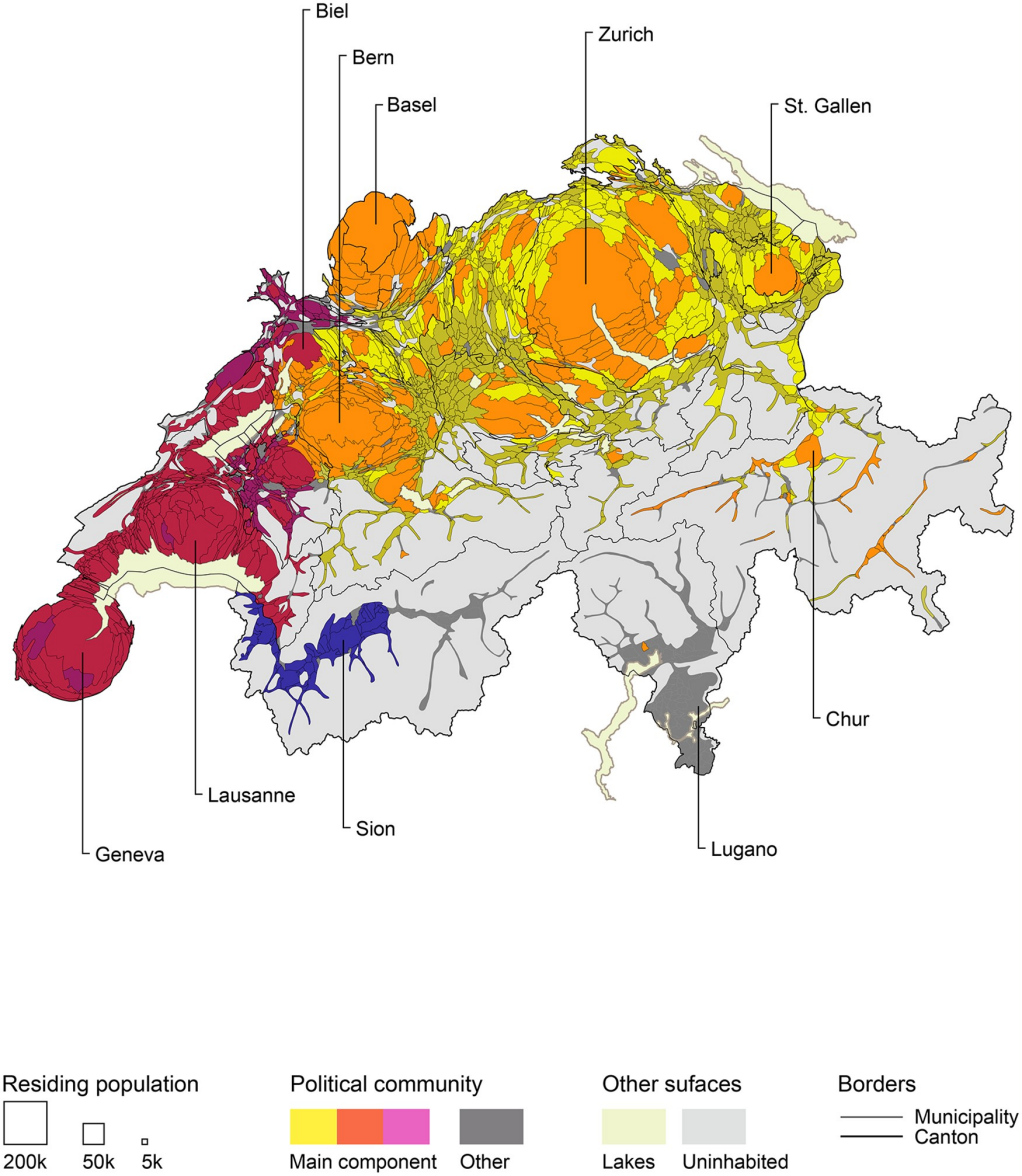
Community partitioning of political agreement above 0.8 *Alpha*, votes called between 1981 and 2015. Residing population size in 2012 census. Cartogram basemap: EPFL-Chôros.

Using higher agreement values overall popular votes, I map communities from the main component of the network discussed above. Fig 4, shows the resulting fifteen political communities that form either regional or inter-regional clusters. To make these results even clearer, I use a *differentiated cartogram* base map so that the size of each area unit is proportional to the size of its residing population while maintaining the surface area of uninhabited regions [88]. I also color code the political communities in relation to their relative position within the networks. Like in the network visualizations I present, colors that are more similar represent clusters that are closer together.

This first map shows the geographic organization of political cleavages between Swiss municipal populations over the last four decades. A combination of four cultural attributes structure political communities: dominant national language, regional particularities, degree of urbanization and global connectivity. Results illustrate how political congruence operates according to two types of relationships between local populations. The first logic consists of topographical articulations, by which populations that are near to one another are more related. Regional political communities such as in Lower Wallis and Romandie give local examples of this contiguous jointure. The second logic offers a complimentary linkage for distant places. This topological articulation appears most clearly between populations of larger German-speaking cities such as Zurich, Bern, Basel, Luzern and St-Gallen, but is also present between certain smaller populations in Romandie. In both cases, the geographic layout of political communities illustrates a certain combination of the two types of spatial articulations.

**Fig 5:**
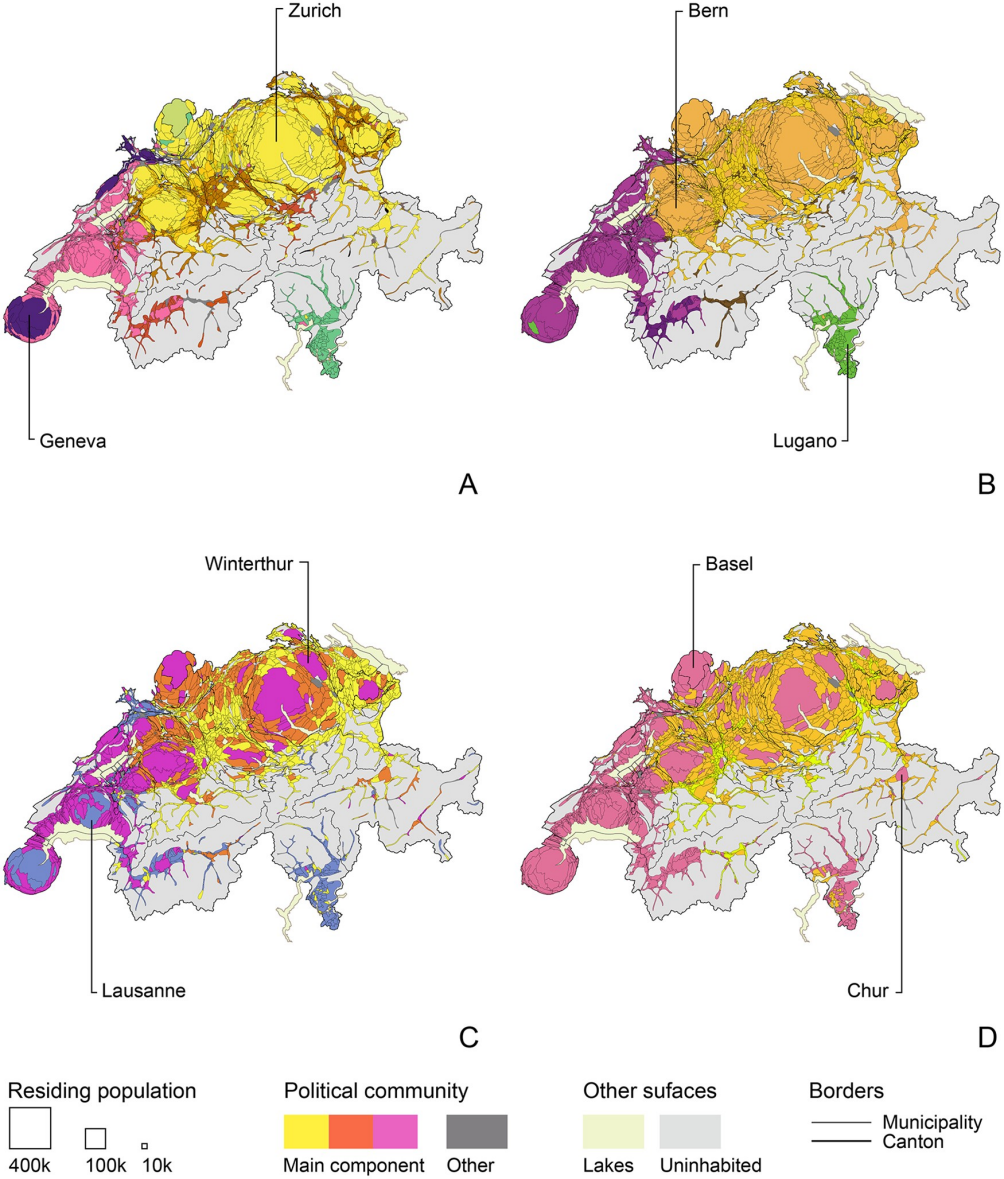
Community partitioning of political agreement above 0.8 *Alpha*, votes called in four sectional periods between 1981 and 2015. (A) 1981-1990. (B) 1991-2000. (C) 2001-2010. (D) 2011-2015. Residing population size in 2012 census. Cartogram basemap: EPFL-Chôros, 2016.

Mapping the community structure for each sectional period allows me to check how that logic evolves and to assess the changing nature of political divides. The four cartograms included in Fig 5 show precisely the extent to which the country’s popular political landscape has engaged in a radical transformation. What is most remarkable is the apparent contiguity of this evolution. The layout of communities shifted from highly localized and partitioned regions in the 1980s to a clear opposition between cohesive linguistic groups in the 1990s. This, again, contrasts with the alignment of major German-speaking cities such as Zurich and Bern with most of the French-speaking and Italian-speaking populations in the 2000s, which culminates into a sharp triptych divide in the first half of the 2010s. Over the past five years, the political alignment of local populations has articulated a two-fold geographical logic. On the one hand, agreement over federal votes points at the importance of localities’ degree of urbanization, splitting German-speaking communities in three clusters: urban, suburban and periurban. On the other hand, French-speaking, Italian-speaking and Romansh-speaking populations expressed strong consensus with each other and with German-speaking urban centers. Cultural divides such as language, which have been believed to be decisive in political processes, are becoming less important than other forms of cultural characteristics such as proximity to urban centers or global connectivity. This latter characteristic is revealed by the higher agreement between certain remote villages and larger cities. Common to those “anomalies” is the presence of historic activities for a global and affluent clientele: thermal baths, ski resorts, international headquarters, global events, luxury goods production, etc. Finally, recent alignments suggest a possible resonance between cultural minorities and large urban centers located within the dominant linguistic group. Such rapid and profound transformations in the geography of political communities call into question the stability of democratic institutions, and highlight the risks of political upheaval in a country considered one of the most politically stable.

This mapping of agreement network communities affords new perspectives on the geographic evolution of popular political cleavages in Switzerland. Yet, the observations I make from the cartograms stem from many factors. Attributes of network visualization, characteristics of the community detection algorithm, the choice of a cartographic base map, the color coding of communities, among others. Because all of those factors are inherent to the heuristic process, I deem it necessary to provide such observations with complementary means, like reporting descriptive characteristics of the networks or visualizing networks into graphs. In the following part, I present yet another way DyAM can be implemented by looking at agreement/disagreement values from the perspective of every single unit of analysis.

### 4.3 Thousands of unique perspectives

Previous parts of the results suggest means to assess the general organization of local populations according to their degree of political agreement over federal popular votes. Another way to look at the evolution of political alignments in those populations is to focus on one municipality at a time. Here, I look at how local populations “move” within the nation’s political network. Again, the combination of a fixed measure of agreement/disagreement and Network Analysis allows yet another relational reading of the data using an egocentric network approach.

From the (N) complete graph of agreement/disagreement, I select one municipal population as the source node of an egocentric network with singular in-degree equaling the number of edges at N-1. To each node, I attribute the dyadic *Alpha* measure linking it to the central node. I then plot the values onto a choropleth of Swiss municipalities and color code them accordingly. I set the gradients to absolute values of the *Alpha*, ranging from dark red at -1, to white at 0, to purple at 1; this facilitates the comparison of egocentric agreement/disagreement values across sectional periods, and between selected municipal populations. In the following section, I report results for three municipal populations, namely the two most populous cities in Switzerland, one German-speaking (Zurich), the other French-speaking (Geneva), and one of the remote populations that disagree most with the two (Unteriberg).

**Fig 6:**
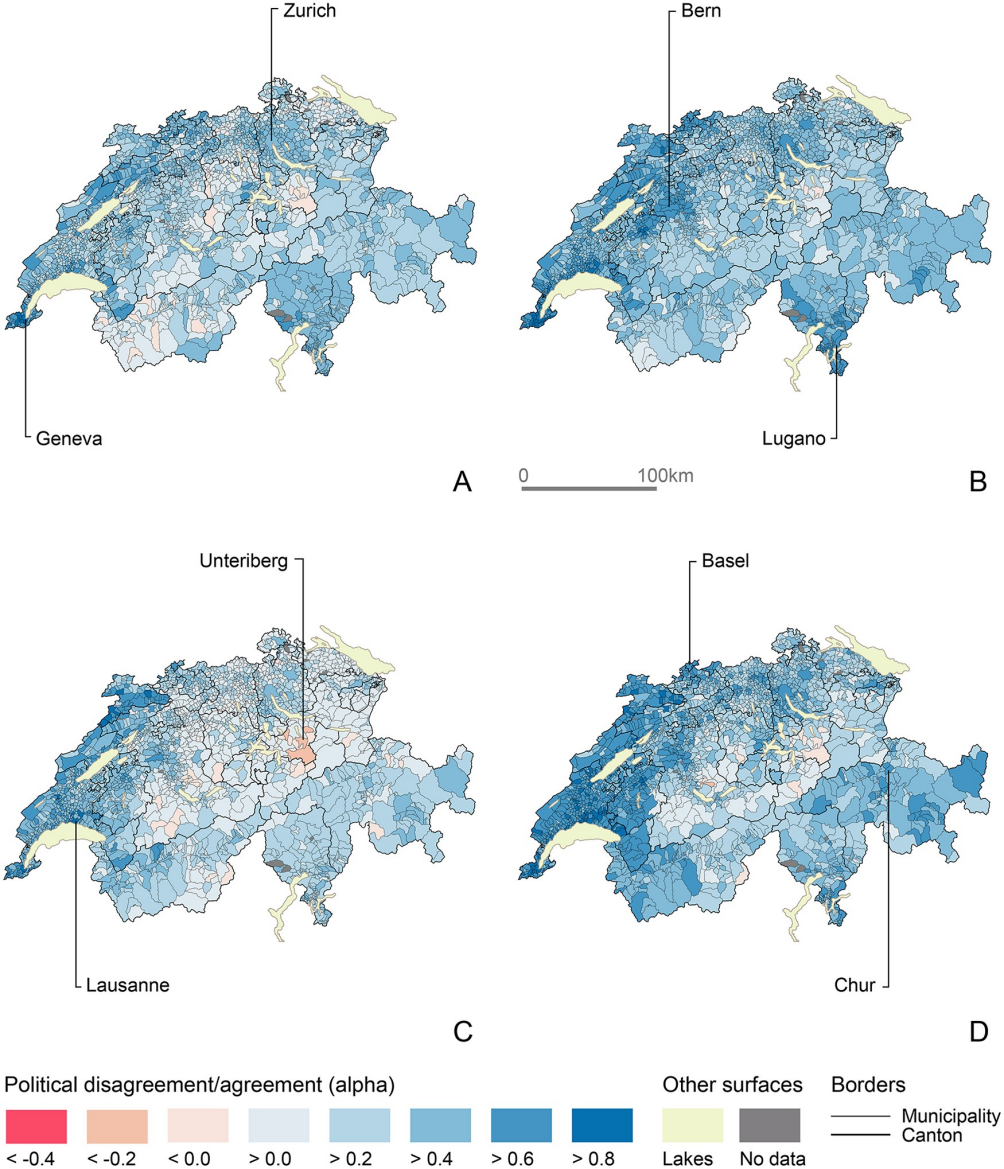
Evolution of popular agreement/disagreement with the municipal population of Geneva. (A) 1981-1990. (B) 1991-2000. (C) 2001-2010. (D) 2011-2015. Choropleth basemap: SFOS, 2016.

By mapping egocentric networks across time provides additional insights on the evolution of mass political congruence in Switzerland. The analysis of specific local populations enables a more subtle representation of political alignments and divides. Fig 6 provides such an example with Geneva. Over the last thirty years, voters in Geneva have sided mostly with populations residing in and around other large cities, the Romand region, Ticino and the Grisons. Political agreement with other populations has increased almost everywhere, following a more antagonizing period in the 2000s. The increase in especially visible in Wallis while political agreement subsists with populations of Central-Switzerland such as Unteriberg, an alpine town of over 2,000 residents.

**Fig 7:**
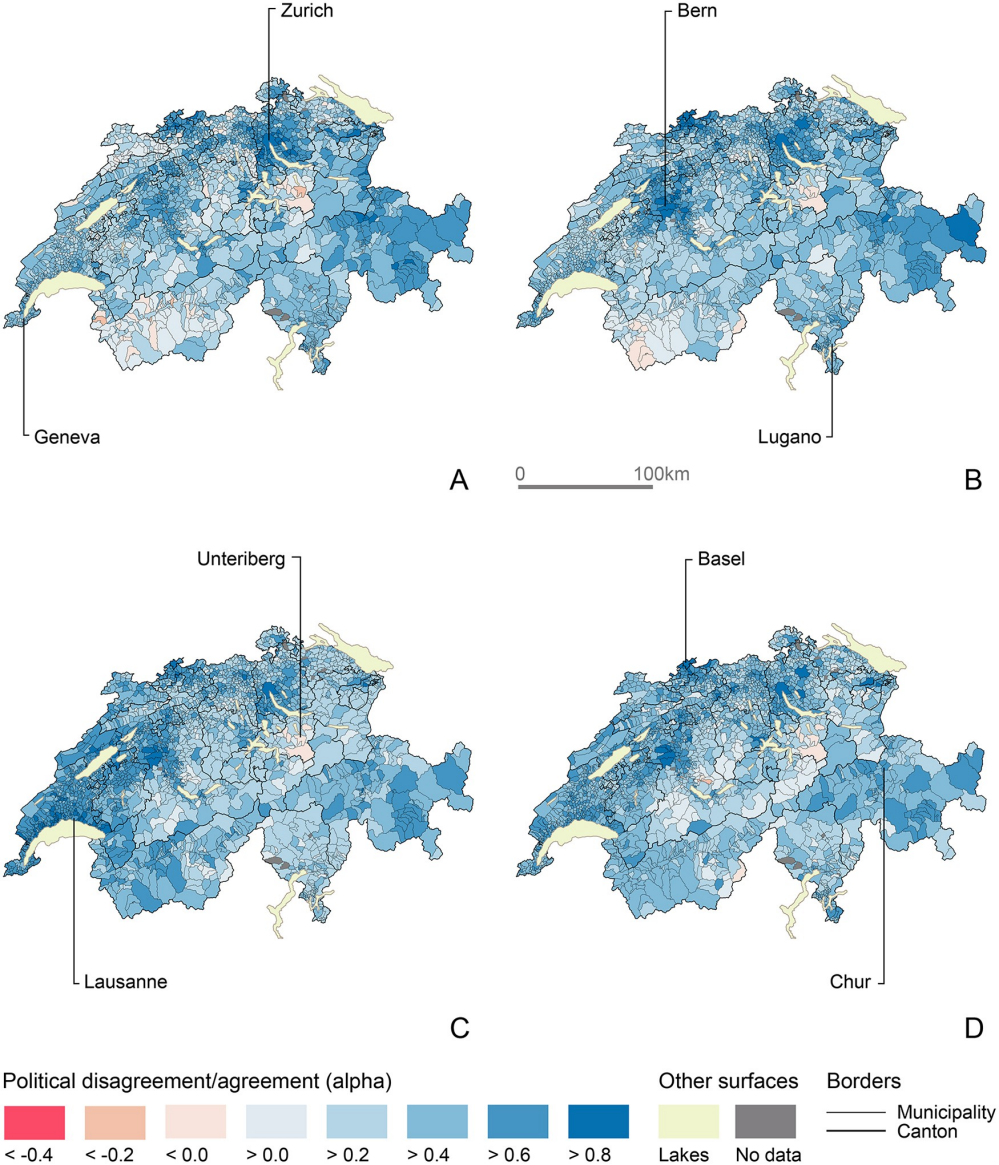
Evolution of popular agreement/disagreement with the municipal population of Zurich. (A) 1981-1990. (B) 1991-2000. (C) 2001-2010. (D) 2011-2015. Choropleth basemap: SFOS, 2016.

I find similar patterns in Fig 7, which displays political disagreement and agreement with the population of Zurich. For the financial center of the country, the most obvious transition occurs between the 1990s and the 2000s when residents of the German-speaking city distanced themselves from Eastern populations and aligned with French-speaking ones. This represents only a brief hiatus before the political convergence of the population of Zurich with those of larger urban centers such as Geneva, Lausanne, Bern, Basel, Luzern, Winterthur and St Gallen. Again, I find political disagreement with voters in Central-Switzerland, in the heart of the canton of Bern and in Upper-Wallis. Across all periods, populations in and around Unteriberg, in Canton Schwyz, are those that disagree the most politically with voters of Zurich.

**Fig 8:**
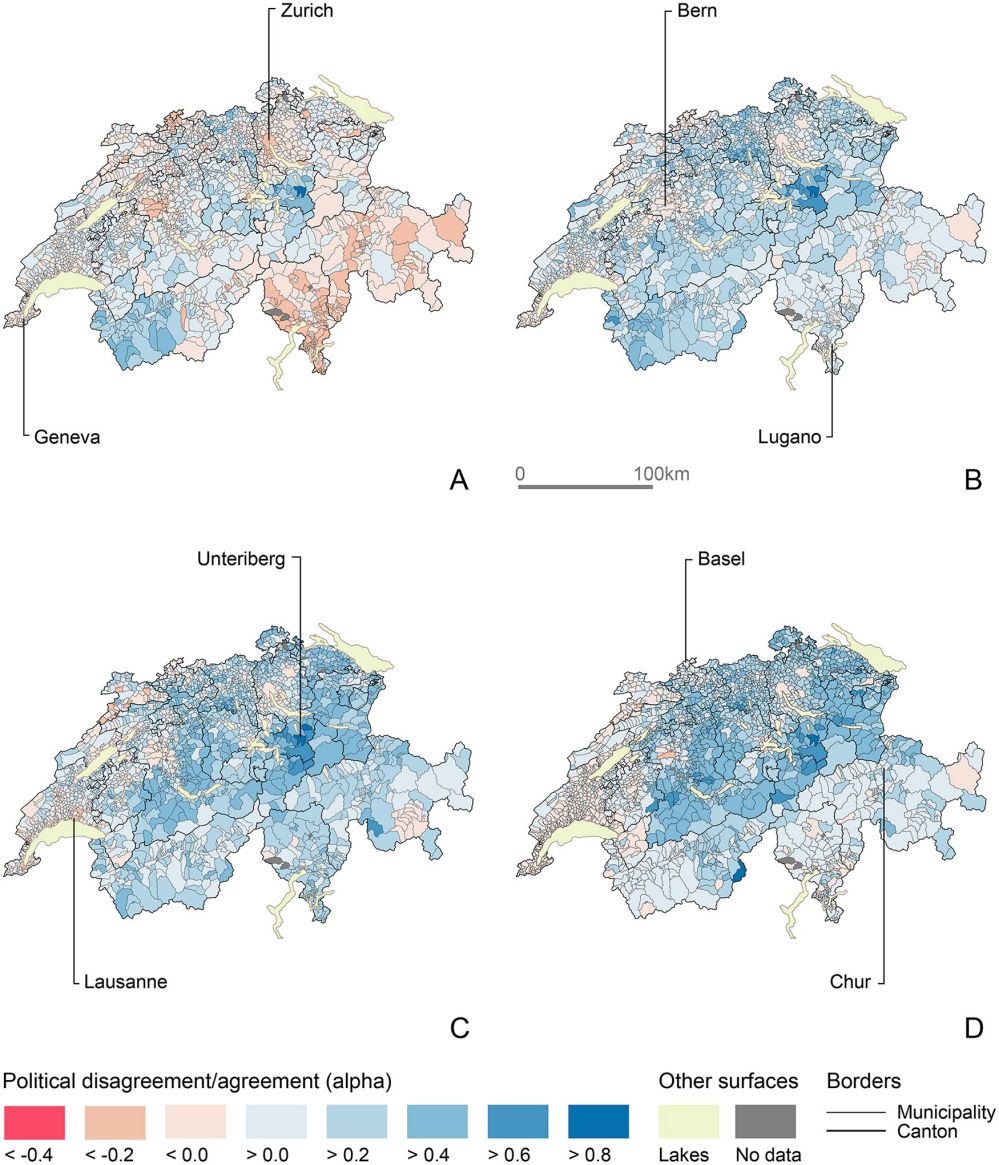
Evolution of popular agreement/disagreement with the municipal population of Unteriberg. (A) 1981-1990. (B) 1991-2000. (C) 2001-2010. (D) 2011-2015. Choropleth basemap: SFOS, 2016.

Because both examples above show the population of Unteriberg to be strongly opposed to possibly the most urban populations of the country—either French-speaking or German-speaking—, I show in Fig 8 which local populations agree and disagree politically with the periurban town. Unsurprisingly, I find voters of Unteriberg disagree mostly with those of major Swiss cities and their surroundings, Ticino and the Grisons. In the 1980s, this population disagrees with most Swiss localities. Subsequently, agreement values tend to increase, especially with other German-speaking populations. Still, in the last sectional period, vote outcomes in Unteriberg remain strongly opposed to larger urban municipalities of Zurich, Winterthur, St Gall and Bern, and with most of the French-speaking populations.

Looking at the evolution of each local population’s agreement/disagreement with voters in other areas provides additional empirical knowledge on Switzerland’s political dynamics. The egocentric networks that result from this approach also make it possible to consider negative *Alpha* values, which I had to discard in the community detection approach detailed above. Such values not only show that certain populations produce antagonizing vote outcomes, but also help to contextualize those that agree on most votes. Moreover, the evolution of specific municipal populations allows us to hypothesize on what outcomes can be expected from future votes. Cautious about the intent to infer on the behavior of individuals, participatory votes still represent survey-like data with very large sample size. To make better sense of the findings DyAM allows, further research should enlight us on how political trends relate to network characteristics such as centrality measures, triads, efficiency and transitivity. To this end, a combination of statistical measures, visual heuristic and the qualitative assessment of local populations’ dynamics offer complementary tools to produce empirical knowledge.

## 5 Reflecting upon topological regionalization of collective political behavior

In this article, I show how political agreement between local populations produces coherent geographic patterns and how these patterns change over time. Results of the DyAM point to an overall convergence of collective vote outcomes; the increasing political congruence between urban centers; the alignment between voters in larger cities and those of linguistic minority regions; the disappearance of language-induced cleavages; and the resilience of the divide between cities, suburbs and remote areas for German-speaking voters. These results concur largely with previous findings and assumptions on the dynamics of Swiss political cleavages. In addition, they hint at many more findings that ought to be explored in future research aimed at unraveling the potential applications of DyAM.

Additional research would also be necessary to solidify these findings. For example, changes in the themes of votes could make some cleavages more salient. Popular initiatives have mostly been backed by Left parties in the past, but are increasingly used by Right parties nowadays. Likewise, changes in partisan support or party politics may affect the geographic evolution of political cleavages. These confounding factors help nuance results of DyAM, but could eventually also be addressed through the method itself. Unlike multivariate approaches, DyAM allows researchers to compare measures of political congruence between local populations. This is also true for other subsets of the data. DyAM could therefore be used to explore whether the themes of the votes are responsible for increasing or decreasing political cleavages between given populations, how party support influences polarization, or if votes should be weighted. Further analyses on the specific application of the *Alpha* would reinforce the research. In this article, I use binary vote outcomes to measure political congruence between local populations. I find that this data reduces measured *Alpha* values by 20% when compared with vote outcomes expressed as ratios. A *paired samples t-test* confirms that the difference is consistent for every dyad in the network. While this does not affect outcomes of this article, variations across levels of measurement would need to be addressed in order to identify strict thresholds of political congruence.

As reported in this article, the DyAM tackles two acute methodological limitations in political research. The first consists in the measure of political disagreement between local populations. Here, the *Alpha*’s intrinsic control for agreement by chance offers such possibility. The second limitation concerns the difficulty in comparing the geographic organization of political communities across time. Contrary to existing approaches, DyAM makes such comparisons possible. As such, the method succeeds in addressing political polarization as a dynamic process, rather than a static state [1]. This capacity to “measure” the depth of political cleavages across time and space is, to my knowledge, unique to DyAM, and offers a substantive contribution to electoral geography and political science.

The implementation of the *Alpha* trough Network Analysis offers additional tools and metrics. I show how egocentric networks provide additional information on the dynamics of political alignments for every single municipal population. Community structures and elementary network parameters such as density, modularity and transitivity give information on the overall systems and its components. Network Analysis thus offers a large range of other metrics, parameters and visualization techniques that I do not showcase in this article. Likewise, ongoing developments in the statistical analysis of network dynamics [89–92] shall be tested on DyAM to identify the effects of monadic and dyadic characteristics of local populations on political cleavages and polarization. Throughout its development, DyAM should largely contribute to answering pressing and important questions in political research. The transdisciplinary nature of network approaches shall also permit findings made with DyAM to connect outside of the disciplinary context in which they are produced. For example, the “geography-blind” patterns that result from DyAM also confront a fundamental assumption of geography: the idea of regionalization as the logic that governs space. In the following part, I wish to discuss how a relational approach to political behavior, which focuses on populations’ similarity, nuances the idea that nearer things are more related. Two figures offer the right material to do so: the topological evolution of political communities over four sectional periods in Fig 5 and the evolution of that Swiss political landscape from the point of view of Zurich in Fig 7.

Space is a complex dimension of society. Addressing such complexity to better understand humans as individuals and as groups, geographers model space on an empirical as well as a conceptual level. In a diagrammatic definition, Lévy speaks of space as set of distance relationships between different social realities. To him, each space holds at least three attributes: a substance, which defines the type of distance considered; a scale, which defines the range of the distance; and a metric, which defines how the distance is measured. Spaces result from the combination of those attributes. For example, contiguous regions are a specific case of spatial configurations: a space in which Euclidean metrics are used to describe both its internal relationships and its limits [93]. Such definition applies to most patterns I find in the first two sectional periods illustrated in Fig 5: we see most political communities during the ‘80s and ‘90s organize into regions that tie contiguous local populations divided mostly by linguistic frontiers. Yet, we also see few political communities that span “across space” and comprise distant or at least non-contiguous populations.

This second type of spatial configuration becomes more obvious in the periods that follow. After 2001, political communities are practically free from linguistic regionalization. For example, one community connects major cities together with most French-, Italian- and Romansh-speaking populations, despite these populations being “not near” one another. Fig 7 illustrates how the population of Zurich is more similar to those in larger cities, French-speaking municipalities and the Grisons, than with denizens of nearer areas of the canton. While both types of spatial configuration exist within each political community, the latter topological format does seem to become a dominant mode of organization in the Swiss political landscape, substituting itself to the former topographical layout. Of course, such considerations do not contradict Tobler’s “first law of geography” altogether [86], but warn us about what “closer” and “related” may mean in terms of spatial relationships. While the idea behind DyAM is mostly inherited from a long-standing tradition in human geography that focuses on relations to identify geographic spaces that are relevant but hard to grasp intuitively, many aspects of the method and its applications shall be refined in subsequent analyses.

The patterns I detect with DyAM suggest that contemporary political cleavages in Switzerland relate more to the global connectivity of local populations than to so-called “urban-rural” or “center-periphery” oppositions. From the findings, I propose a new typology to explain political divides. I call “extrovert” clusters where local populations have interacted with foreigners for a long time. “Introvert,” in opposition, are clusters where local populations have had fewer direct interactions with otherness. In between, “selective” populations live closer to “extrovert” locations, but foreigners are unlikely to be found in their neighborhoods. In Switzerland, this model explains why distant populations may repeatedly produce similar outcomes over federal votes. It matches the “anomalies” I detect with the method, as well as the convergence of major cities with linguistic minorities, and the clustering of German-speaking populations across suburbs and remote areas. Moreover, such patterns resemble those observed in recent elections and referendums across Northern countries. With this framework, I wish to anchor the study of mass political polarization in the daily and multidimensional dynamics of human interactions and exchanges, and the complexity of relational space. I believe that DyAM offers a step towards such theoretical considerations. In the following, I wish to discuss three issues that need to be addressed with DyAM in order further pursue this research agenda.

The first issue concerns the difficulty to interpret measures of agreement/disagreement. This article attempts to measure political agreement/disagreement between local populations over multiple votes. To do so, I use a singular set of data, municipal outcomes over Swiss federal participatory votes. Direct democracy in Switzerland represents a singular political institution with very few possible cases to compare [94]. While more or less frequent usage of popular votes occur in certain places, such as California or Italy, the Swiss direct democratic system holds a unique importance within the daily life of Swiss people. Krippendorff’s *Alpha* is a measure of reliability designed to check how coders agree or disagree over content. The formula is primarily used in Content Analysis with few applications in other methodological contexts. As shown in the present study, the *Alpha* finds many practical and conceptual compatibility with vote outcomes and the study of political cleavages. Yet, the lack of reference work makes it difficult to give context the measures of agreement/disagreement. Further research could therefore focus on comparing agreement/disagreement over dominant political themes or juridical type of votes. Likewise, outcomes should be contrasted with those for participatory votes in California. Looking at agreement/disagreement over a series of elections could also prove valuable, especially in opening the method to other case studies, despite the infrequent occurrence and general complexity of such democratic decision-making process. From a methodological point of view, the full potential application of the *Alpha* to political congruence remains to be tested, and should therefore be compared to other metrics. Instead of computing agreement/disagreement from binary vote outcomes, further research should attempt to similar work using continuous or ranked values, and see how they affect results. Similarly, because of the *Alpha*’s capacity to measure agreement/disagreement for any number of populations, it could be used for groups with more than two sets of voters to depict contrast within states or regions. DyAM could then be used across levels to assess political agreement/disagreement between individual voters, local populations, party members, political groups and organizations, thus offering a methodological solution to the investigation of micro-macro relationships in political dynamics.

Such exploitation of DyAM to generate ecological measurements points a second issue, which concerns the analysis of collective political behavior using aggregated data for vote results. In the present study, I use results of federal participatory votes to identify regional and inter-regional political divides and alignments between localities. For each vote object, the data concerns the number of cast ballots and the proportion of ballots in support. Results I present can only illustrate the collective behavior of active voters, thus accounting for a large share of municipal residents. Yet, Swiss municipalities—communes—present very heterogeneous territorial and demographic characteristics. They vary greatly in terms of land area, landscape, usage, industry, job market, infrastructure, population size, religious affiliation, language, age and ethnic composition. Some cities house up to forty percent non-national residents, while border regions have strong daily influx of cross-border workers. Switzerland’s repute for beautiful landscapes, winter activities and historical architecture attracts millions of tourists each year. The country’s larger cities and alpine regions have become international hubs for affluent visitors and diplomats. Secondary houses are common in those areas for either recreational or work-related purposes, and Swiss people spend much of their time outside the municipality where they have registered their main residence [95]. Likewise, agreement/disagreement measures that I report rather illustrate local contexts rather than individual political attitudes, for it is unlikely that voters remain the same from one vote to the next, especially over a 34-year period. All those conditions—those of a “real world”—limit the possibility to offer non-schematic interpretation of large geographic patterns built from binary collective vote outcomes. Yet, the coherence between the results, the demographics of Swiss local populations and the country’s political dynamics suggest the method is able to model this relationship at a sufficient degree of precision to produce meaningful empirical knowledge. DyAM thus offers a precious contribution to the geographic study of political cleavages across local populations. In a “data-driven world,” such a timely contribution helps reconsider common assumptions about the regionalization of political behaviors. The increase importance of Big Data, and the ensuing necessity to anonymize individuals from multilayered datasets could surely benefit from this method and its eventual development for even smaller levels of aggregation.

Finally, another set of issues with the method concerns characteristics of the techniques I combine. An important contribution of the *Alpha* holds to its capacity to account for disagreement as a distinct measure from low agreement. Because an agreement/disagreement by chance coincides with *α* = 0, I keep the metrics’ value space from -1 to 0 for disagreement and from 0 to +1 for agreement. While negative values are common in real-life social network, their usage in Network Analysis remains difficult. This, because many community detection algorithms and network visualization algorithms cannot handle negative values. In the present study, I use both types of algorithms, which limits my use of negative *Alpha* values. To overcome this limitation, I avoid the use of algorithmic calculations by mapping out “egocentric networks,” which portray political agreement/disagreement for each sectional period from the perspective of selected case studies. Hence, the study I present here offers a departure point to a new departure for the geographical analysis of political cleavages, not its conclusion. In that sense, DyAM offers to measure political disagreement but—like for positive values in the *Alpha*—still lacks of necessary empirical evidences to qualify what “disagreement” means in terms of measured *Alpha* values. In this article, I mostly rely on traditional visualization techniques I borrow from Network Analysis and cartography in order to interpret outcomes of DyAM. As in any visualization, I adjust the parameters in order to offer the greatest readability for the data. Although I have little control over the spatialization algorithms used to visualize networks into graphs, I am able to adjust the orientation, the transparency and the coloring of edges and nodes to provide more coherent illustrations. For example, in order to coherently color code the communities detected in each graph, I use a color wheel. For each network, I carefully choose the position and the orientation of the color wheel so that communities’ colors match across different sectional periods. For the lack of a better technique, I thus color-code political communities in order to avoid the use of random colors, which would lessen the readability of graphs and cartograms. Similarly, I use “differentiated cartograms” because they provide a more pertinent metric of space—that of municipalities’ populations size—while also making it easy to navigate the map for someone familiar with the geography of Switzerland.

These issues illustrate the challenges of transdisciplinary analyses. As such, DyAM contributes to a growing body of research on the definition of spatial boundaries stemmig from social connectedness [85, 96]. In a like manner to the study I present, this work outlines social processes as inherently spatial, define spatial boundaries in terms of social relationships, and ground their method in network analysis.

## 6 Conclusion

This study provides new methodological grounds to measure the evolution of political cleavages at a macroscopic level and test hypotheses on the polarization of voters. A geographic analysis of municipal outcomes over Swiss federal participatory votes called between 1981 and 2015 points to the political convergence of local populations; the de-regionalization of political polarization; the alignment the voting majority living in larger cities with those from national linguistic minority regions; the erosion of the long-standing “linguistic divide”; and the resilience of new alignments and divides between local populations in larger cities, suburbs, remote areas, cosmopolitan villages and minority cultural regions. Behind those findings, the method of *Dyadic Agreement Modeling*, which I present in this article, highlights the dynamic nature of political polarization. Results illustrate the emergence of strong topological spatial relationships over political matters, matching an intuitive understanding of current social dynamics as increasingly built upon material and immaterial networks. The methodological as well as empirical contributions I make here should prove useful to policy-makers, political strategists together with spatial planners, and contribute to renewing an interest for the ecological analysis of political behavior and polarization.
